# Autoimmune Post-Herpes Simplex Encephalitis

**DOI:** 10.15844/pedneurbriefs-30-3-6

**Published:** 2016-03

**Authors:** Ana B. Chelse, Leon G. Epstein

**Affiliations:** 1 Division of Neurology, Ann & Robert H. Lurie Children's Hospital of Chicago, Chicago, IL; 2 Departments of Pediatrics and Neurology, Northwestern University Feinberg School of Medicine, Chicago, IL

**Keywords:** Herpes Simplex Virus, Encephalitis, Autoimmunity

## Abstract

Investigators at August Pi Sunyer Biomedical Research Institute and University of Barcelona performed a prospective surveillance study in 14 patients with immune-mediating relapsing symptoms of post-herpes simplex encephalitis (HSE).

Investigators at August Pi Sunyer Biomedical Research Institute and University of Barcelona performed a prospective surveillance study in 14 patients with immune-mediating relapsing symptoms of post-herpes simplex encephalitis (HSE). The study aimed to compare the clinical and immunologic features of HSE in teenage and adult patients with those of young children. Groups were divided into 6 young children aged 6 to 20 months (median, 13 months) and 8 teenager and adult patients aged 13-69 years (median, 40 years). All patients underwent repeat serum and CSF HSV PCR and MRI of the brain. CSF was also sent for antibodies to cell surface and synaptic proteins, including NMDA and GABA. Repeat CSF was negative for HSV in all patients with symptoms suggestive of disease recurrence. Symptoms concerning for recurrence differed between groups. Adults and teenagers had longer delays in diagnosis (85 days, range 17-296) when compared to children (4 days, range 0-33, p= 0.037). All Children <20months of age developed choreoathetosis (6/6 vs 0/8, p < 0.01) and decreased level of consciousness (6/6 vs 2/8, p < 0.01) in association with IgG antibodies against NMDA receptor subunits (6/6). In contrast, the teenage and adult patients did not develop choreoathetosis. 3/8 older patients presented with symptoms resembling acute viral relapse; 5/8 developed symptoms while in rehabilitation initially attributed to disease recrudescence. 7/8 patients developed severed neuro-psychiatric and behavioral symptoms. 1/8 developed blepharospasm. 5/8 had CSF antibodies against NMDA receptor and 3/8 against unknown neuronal cell surface proteins. In 5/6 patients the MRI showed new areas of contrast enhancement during relapse that decreased after immunotherapy. 1/8 patients showed spontaneous recovery and other 7/8 were treated with immunotherapy. Therapies included steroids alone, steroids with IVIG, or steroids with IVIG, and plasma exchange. Immunotherapy was beneficial in 100% of patients treated. [1]

COMMENTARY. The current literature supports the hypothesis that HSE triggers auto-antibodies against synaptic proteins, like N-methyl-D-aspartate receptor (NMDAR) and other brain autoimmunity [2, 3, 4]. The current study adds important information to the theory of auto-immunity after HSE. It also shows that symptoms of HSE relapse and subsequent brain autoimmunity differs between children and older patients. Specifically, teenage and adult patients should be monitored closely as they tend to present with milder symptoms early in their relapse that may be difficult to distinguish from exacerbation of baseline deficits. Physicians should assess these patients for both recurrent HSV infections as well as antibodies against synaptic proteins. The authors report an excellent response to immunotherapy; larger placebo-controlled trials are necessary to study the benefit of immunotherapy in prevention and treatment of immune-mediated relapse. Additionally, given the varying symptoms that were present during relapse between age groups, multicenter placebo-controlled studies would help to better characterize the clinical, immunological and neuro-radiographic findings in immune-mediated post-HSE relapse.
